# Circular RNA *Pde4dip* regulates myogenesis by interacting with *Zfp143* mRNA: a novel regulatory axis

**DOI:** 10.1080/15476286.2025.2583576

**Published:** 2025-10-31

**Authors:** Suman Singh, Arundhati Das, Gaurahari Sahoo, Amaresh Chandra Panda

**Affiliations:** Institute of Life Sciences, Nalco Square,Bhubaneswar, India

**Keywords:** RNA-RNA interactions, circular RNA, mRNA regulation, muscle cells, C2C12, myogenesis, microRNA

## Abstract

Many transcriptional and post-transcriptional regulators tightly regulate skeletal muscle myogenesis, including recently discovered circular RNAs (circRNAs). In this study, we used a crosslinking-based sequencing method called CLiPP-Seq, in which we performed AMT-mediated Cross-Linking of RNA-RNA duplexes followed by Poly(A) RNA Pulldown and sequencing to identify mRNA-interacting circRNAs in the mRNA samples of mouse C2C12 myoblasts and myotubes. BLAST analysis of the circRNAs with mRNAs identified their potential interacting partners. Interestingly, silencing of the circular RNA *Pde4dip* (*circPde4dip*) altered the target mRNA *Zfp143* (zinc finger protein 143) expression and suppressed the differentiation of C2C12 myoblasts into myotubes. In summary, we identified unexplored mRNA-interacting circRNAs and their possible functions in muscle cell differentiation, specifically the *circPde4dip-Zfp143* mRNA interaction regulating myogenesis.

## Introduction

Skeletal muscle is the largest organ responsible for movement and metabolism [1]. Skeletal muscle is generated and maintained by the proliferation and differentiation of myogenic stem cells [2]. A range of transcription factors, including Pax3, Pax7, MyoD, Myf5, Myogenin, and Mrf4, are pivotal in regulating gene expression during the myoblast proliferation and differentiation into myotubes [3]. In addition to the well-studied transcription factors, recent research has suggested that noncoding RNAs, such as microRNAs (miRNAs), long noncoding RNAs (lncRNAs), and recently discovered circular RNAs (circRNAs), also play significant regulatory roles [4,5]. CircRNAs have elicited considerable interest owing to their extraordinary stability, evolutionary conservation, tissue-specific expression, and altered expression in various developmental or disease states [6]. For instance, their expression levels are substantially altered during ageing and muscle cell differentiation [7]. The involvement of circRNAs in myogenesis has only become apparent in recent years, and many studies have demonstrated that circRNAs regulate myogenesis mainly by interacting with miRNAs and RBPs. While most studies have reported interactions between circRNAs and miRNAs or proteins, no clear picture of the direct circRNA-mRNA interaction has emerged so far [8].

Previously, we reported hundreds of circRNA-mRNA interactions in Beta-TC-6 cells, 2-days differentiated C2C12 cells, and HeLa cells [9]. Another report demonstrated that *circZNF609* directly binds to *CKAP5* mRNA, increasing CKAP5 translation, regulating microtubule function in cancer cells, and supporting cell-cycle progression [10]. Furthermore, to understand the role of mRNA-interacting circRNAs during myogenesis, we performed AMT-mediated crosslinking of transcripts expressed in C2C12 myoblast and 6-days differentiated myotube cells, followed by mRNA-hybrid complex pulldown and high-throughput sequencing called CLiPP-Seq [9]. We identified approximately 700 mRNA-interacting circRNAs with varying expression during myoblast differentiation. In this study, we investigated one such mRNA-interacting circRNA, *circPde4dip*, which most prominently interacts with *Zfp143* mRNA. We demonstrated that silencing *circPde4dip* increased expression of its interacting *Zfp143* mRNA and ZFP143 protein and reduced C2C12 myoblast differentiation into myotubes. This study revealed a previously unknown regulatory role for circRNAs, which directly interact with mRNAs during myogenesis and emphasized their direct effects on mRNA expression.

## Results

### CLiPP-Seq identified mRNA-interacting circRNAs in C2C12 myoblasts and myotubes

**Figure 1.**
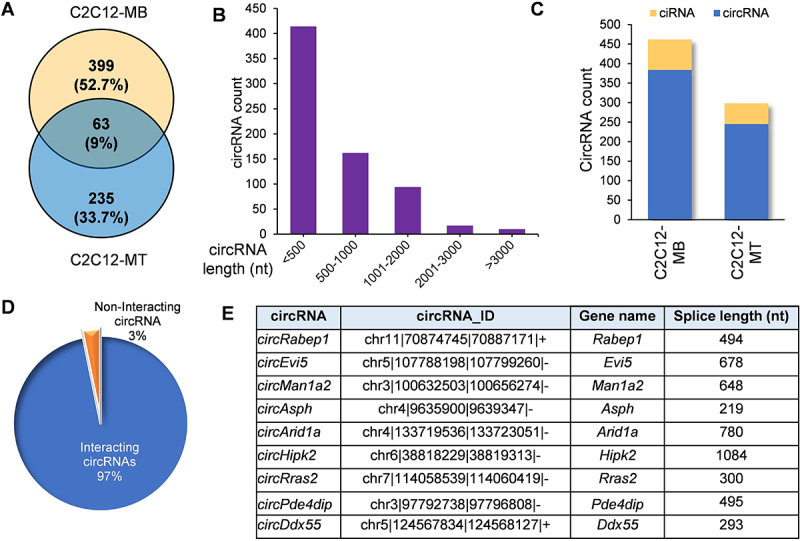
The mRNA-interacting circRNAs identified in C2C12-myoblast and myotube by CLiPP-Seq and their characteristics. (A) Venn diagram showing circRNAs identified in C2C12 myoblast and myotube-specific CLiPP-seq data sets. (B) Bar graph representing the splice lengths of total circRNAs identified in C2C12 myoblast and myotubes. (C) Bar graph showing exonic and intronic circRNAs identified in both datasets. (D) Pie chart showing the number of circRNAs interacting with mRNAs identified in blast analysis of CLiPP-seq samples. (E) Selected list of mRNA-interacting circRNAs in C2C12 myoblasts and myotubes.

To understand the expression of mRNA-interacting circRNAs during myogenesis, C2C12 myoblasts, and 6-day-differentiated C2C12 myotubes were used for AMT-treated CLiPP-sequencing. Analyzing the CLiPP-seq reads with the CIRCexplorer2 annotation, we identified 697 mRNA-interacting circRNAs in myoblasts and myotubes combined (Supplementary Table S1, Figure 1(A)). A majority of the circRNAs had a splice length of 500 nucleotides, while a few of the identified circRNAs had a splice length of > 1 kb (Figure 1(B)). circRNAs were derived from mouse chromosomes in an unbiased manner, represented along the mouse (mm10) cytoband (Supplementary Figure S1A). These data also showed that 70% of circRNAs were derived from exons, and only a part of the remaining fractions corresponded to circular intronic RNAs (ciRNAs) (Figure 1(C)). We also found that most circRNAs in CLiPP-seq were derived from genes with fewer than ten exons (Supplementary Figure S1B). Few host genes produced multiple circRNAs, but nearly all host genes were capable of producing one or two circRNAs (Supplementary Figure S1C). BLAST analysis identified that about 97% of circRNAs had complementarity with mRNAs in C2C12 CLiPP-seq datasets (Supplementary Table S2, Figure 1(D)).

### Characterization of differentiation-associated mRNA-interacting circRNAs

**Figure 2.**
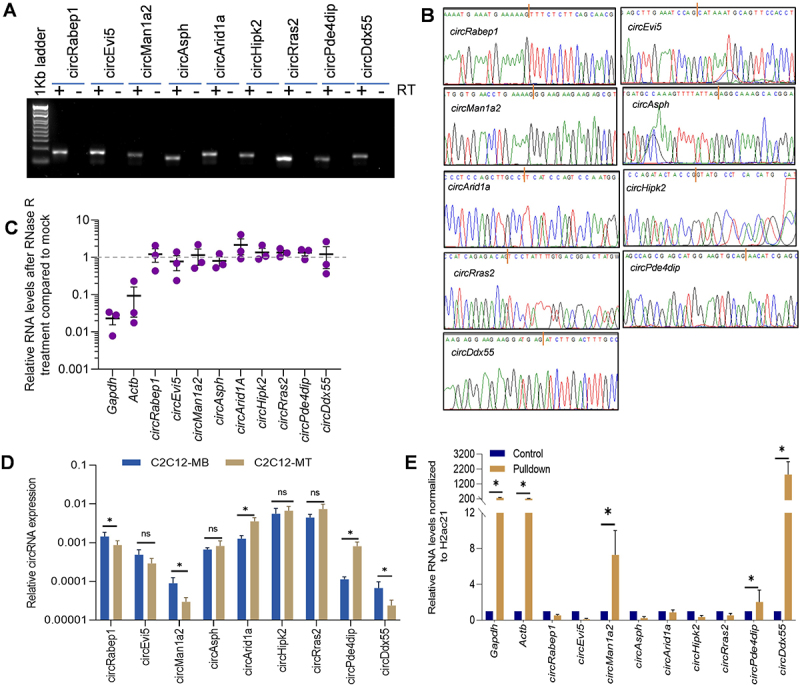
Validation of circRNAs and their enrichment in mRNA pulldown. (A) Visualization of RT/no-RT pcr amplicons in SYBR green-stained 2% agarose gel. (B) The chromatogram shows the circRNA sequence, and the red bar specifies the junction sequences. (C) Interleaved scatter plot showing qRT-PCR analysis after RNase R treatment. (D) RT-qPCR analysis showing the expression pattern of circRNAs in C2C12 myoblasts compared to C2C12 myotube stage. (E) RT-qPCR analysis showing enrichment of *circPde4dip*, *circDdx55*, and *circMan1a2* along with control *Gapdh* and *Actb* mRNAs upon oligo d(T) mediated mRNA pulldown.

From the CLiPP-seq data, we shortlisted a few circRNAs based on certain criteria, including their splice length of < 1.5 kb, exon count of < 5, and abundance in C2C12 myoblasts or myotube cells (Figure 1(E)). We used divergent primer pairs across the backsplice junction to validate the expression of mRNA-interacting circRNAs in C2C12 cells by PCR (Figure 2(A)). As shown in Figure 2(B), purification of the amplified PCR products and Sanger sequencing proved that only the backsplice junction sequences of target circRNAs were specifically amplified. We further subjected the total RNA isolated from C2C12 cells t to RNase R treatment, as it is well known that circRNAs are resistant to exonuclease activity owing to lack of free ends. Linear *Actb* and *Gapdh* mRNAs were significantly degraded upon RNase R digestion, whereas the selected mRNA-interacting circRNAs were immune to degradation, confirming their identity as circular transcripts (Figure 2(C)).

### mRNA-interacting circRNAs are differentially expressed during myogenesis

Proliferating C2C12 myoblasts and 4-days differentiated C2C12 myotubes were used to analyse the differential expression of selected circRNAs. Microscopic images and upregulation of myogenesis markers confirmed the differentiation of C2C12 myoblasts into myotubes (Supplementary Figure S2A, B). Interestingly, some of the selected mRNA-interacting circRNAs, including *circDdx55*, *circArid1a*, *circMan1a2*, *circPde4dip*, and *circRabep1* showed significant differential expression patterns in C2C12 myotubes compared to myoblasts (Figure 2(D)). Furthermore, we performed a poly(A) RNA pulldown assay using oligo-dT beads with total RNA from 2-days differentiated C2C12 myotubes, followed by enrichment analysis to check the interaction of circRNAs with poly(A) RNAs/mRNAs. Notably, several circRNAs, including *circPde4dip*, *circDdx55*, and *circMan1a2*, along with *Gapdh* and *Actb* mRNA, were significantly enriched compared to the input samples, confirming the specific pulldown of poly (A) RNAs and their interacting circRNAs (Figure 2(E)). As *circPde4dip* expression showed the highest upregulation in myotubes compared to myoblasts and significant enrichment in poly (A) RNA pulldown (Figure 2(D,E))we selected *circPde4dip* to further extrapolate its role during C2C12 differentiation.

### circPde4dip is stably expressed and interacts with multiple mRNA targets

**Figure 3.**
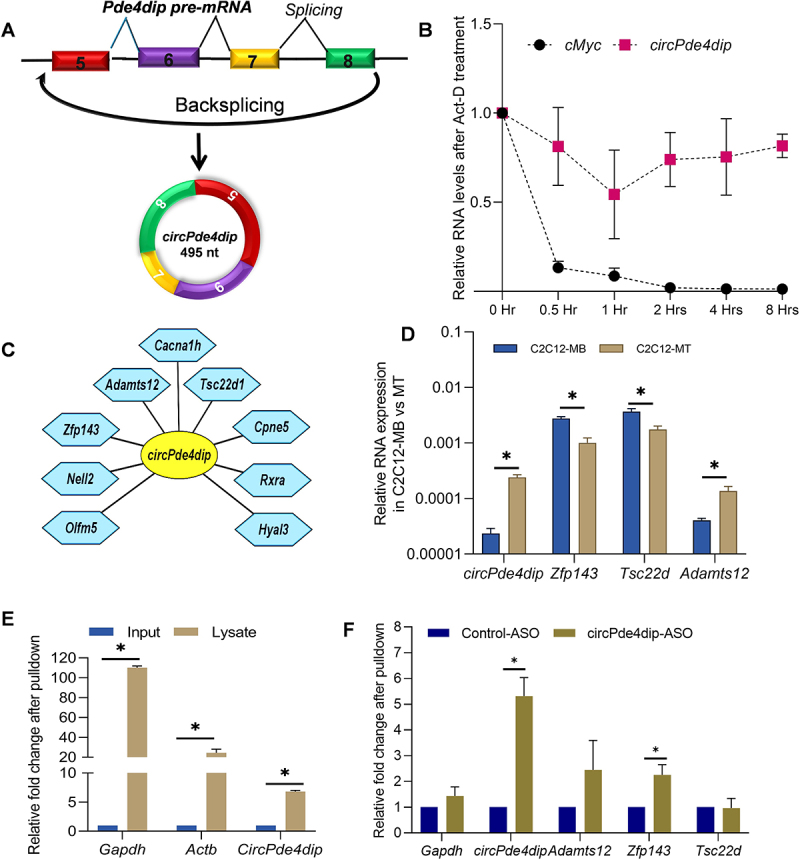
Validation of *circPde4dip*-interacting mRNA expression in C2C12. (A) Schematic representation of circPde4dip biogenesis via backsplicing (B) RT-qPCR analysis showing relative Fold change in *circPde4dip* expression compared to linear *Myc* mRNA after actinomycin D treatment. (C) Cytoscape showing *circPde4dip*-specific mRNA targets based on sequence complementarity. (D) RT-qPCR analysis showing the differential expression of *circPde4dip-*interacting mRNAs in C2C12 myoblasts compared to myotubes. (E) RT-qPCR analysis showing the enrichment of *circPde4dip* in poly(A) pulldown samples compared to input cell lysates of 2-days differentiated C2C12 cells. (F) RT-qPCR results showing enrichment of *Zfp143* mRNA along with *circPde4dip* upon aso pulldown.

Back splicing of *Pde4dip* pre-mRNA (ENSMUSG00000038170) produces a ~ 495-nucleotide exonic circular *Pde4dip* RNA (Figure 3(A), Supplementary Figure S3A). Analysis of the stability of *circPde4dip* in 2-days differentiated C2C12 cells with actinomycin D treatment indicated that *circPde4dip* remained stable until 8 h post-treatment, while *Myc* mRNA progressively degraded within an hour (Figure 3(B)). We analysed the BLAST data to identify several mRNAs that interact with *circPde4dip* in C2C12 cells, as depicted in Cytoscape (Figure 3(C), Supplementary Figure S3B). Since *circPde4dip* was enriched in C2C12 myotubes in the CLiPP-seq data, we checked the expression pattern of its interacting mRNAs as well in C2C12 myoblasts and myotubes. RT-qPCR analysis confirmed reduced expression of *circPde4dip-*interacting zinc finger protein 143 (*Zfp143)* and *Tsc22d* mRNA in myotubes compared to myoblasts (Figure 3(D)). Acknowledging the potential for false-positive results in poly(A) pulldown from total RNA due to mere sequence complementarity, we performed a native pulldown assay on 2-days differentiated C2C12 cell lysates. Notably, enrichment of *circPde4dip* along with *Gapdh* and *Actb* mRNA from poly (A) pulldown in cell lysates further indicated a likely interaction with target mRNAs (Figure 3(E)). Further, using the inverse approach, *circPde4dip* was pulldown in 3-days differentiated C2C12 cell lysates using biotin-tagged antisense oligos (ASO) targeting the backsplice junction sequence, followed by RT-qPCR analysis, which showed significant enrichment of *circPde4dip* along with the target *Zfp143* mRNA, indicating direct base-pairing with *circPde4dip* (Figure 3(F)).

### circPde4dip regulates Zfp143 expression and promotes myogenesis in C2C12 cells

**Figure 4.**
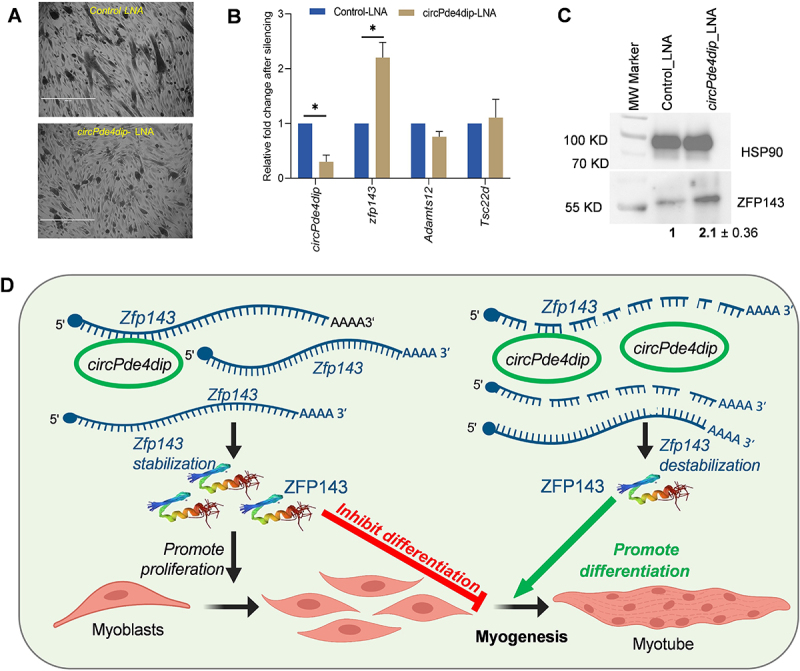
Regulation of C2C12 differentiation by *circPde4dip*. (A) The phase-contrast image showing C2C12 myotubes, 72 hr post-treatment with control lna and *circPde4dip* lna. (B) RT-qPCR analysis showing relative Fold change in *circPde4dip* expression along with target mRNA expression after silencing. (C) Western blot showing ZFP143 expression compared to control HSP90 expression after *circPde4dip* silencing. (D) Possible mechanism of C2C12 differentiation mediated by *circPde4dip* interaction with *Zfp143*. In the myoblast stage the inhibition of C2C12 cell differentiation is driven by the presence of *Zfp143* mRNA which encodes ZFP143 transcription factor responsible for maintaining the cells at the myoblast stage by promoting proliferation. In the myotube stage, *circPde4dip* destabilizes its interacting partner *Zfp143* mRNA, leading to reduced expression of ZFP143 and promoting myotube formation.

Since *circPde4dip* expression was upregulated at the myotube stage, we hypothesized that *circpde4dip* could regulate C2C12 muscle cell differentiation. We silenced *circPde4dip* using a backsplice junction-specific GapmeR and allowed it to differentiate for three days. Phase-contrast images showed reduced myotube formation upon *circPde4dip* silencing compared to control C2C12 cells (Figure 4(A)). Knockdown of *circPde4dip* led to a significant increase in *Zfp143* mRNA expression, whereas *Tsc22d* and *Adamts12* mRNA expression was not affected, indicating that the regulation of myogenesis by *circPde4dip* could be mediated through *Zfp143* (Figure 4(B)). Further, we observed that ZFP143 protein expression was upregulated upon *circPde4dip* silencing, indicating direct regulation of ZFP143 by *circPde4dip* (Figure 4(C)). Furthermore, to rule out miRNA-mediated indirect regulation of *Zfp143* mRNA, we performed miRNA analysis of *circPde4dip* targets and, interestingly, found that *Zfp143* mRNA was not targeted by any of the *circPde4dip*-associated miRNAs (Supplementary Figure S3C, D). Using bimolecular structure predictions and pulldown assays, we proved that circRNAs might directly hybridize with mRNAs to form RNA duplexes (Supplementary Figure S4). Furthermore, to further evaluate the potential influence of *circPde4dip* on proliferation via ZFP143, we performed *circPde4dip* knockdown and evaluated cell proliferation. Interestingly, *circPde4dip* knockdown resulted in an increased C2C12 cell proliferation along with a moderate increase in the cell cycle marker *Ccnd1* mRNA (Supplementary Figure S5). Together, our results demonstrated that increased expression of ZFP143 upon *circPde4dip* silencing could promote the maintenance of cellular stemness and proliferative capacity, thereby preventing myotube formation (Figure 4(D)).

## Discussion

CircRNAs have been proven to be key regulators of the pre-mRNA splicing machinery, transcription, translation, and protein functions by interacting with target miRNAs or RBPs. Given the diverse roles of circRNAs, there has been a significant increase in reports suggesting their role in skeletal muscle differentiation [5]. The ability of circRNAs to regulate myogenesis has been mostly limited to their miRNA-sponging activity, with a few reports on circRNA-RBP interactions in myogenesis. The interplay between circRNAs and mRNAs remains largely unexplored. In this study, we investigated the circRNA-mRNA crosstalk in regulating myogenesis.

Recent shifts in research highlight the role of direct interaction between circRNAs and mRNAs, evidenced by a novel finding showing circZNF609 binds to CKAP5 mRNA and thus plays an essential role in regulating microtubule formation and tumorigenesis [10]. Previously, we reported the direct interaction between circRNAs and mRNAs in HeLa, βTC6, and C2C12 cells [9]. Another study report on circRNA-mediated mRNA decay, further strengthens our hypothesis, in which the circRNA-mRNA interaction brings the exon-junction complex (EJC) into proximity with the 3’UTR, leading to EJC-dependent nonsense-mediated mRNA decay (NMD) [11]. From our BLAST analysis, we understand that most circRNA interactions occur in the UTR region of mRNAs, highlighting their potential regulatory significance [9]. This study is our further attempt to understand the differential roles of mRNA-interacting circRNAs in muscle cell differentiation. From the CLiPP-Seq data, a large number of mRNA-interacting circRNAs were identified in C2C12 myoblasts and myotubes (Figure 1). Furthermore, from the BLAST analysis, we found that 97% of the circRNAs interacted with mRNA. From the pulldown assays, we determined that *circPde4dip* was significantly enriched with linear mRNAs, and from the differential expression analysis we found *circPde4dip* to be an interesting target, as it was significantly upregulated during C2C12 cell differentiation (Figure 2). Although many mRNAs were predicted to be complementary to *circPde4dip*, only *Zfp143* mRNA was significantly enriched upon *circPde4dip* pulldown (Figure 3).

Consistent with the reported findings, we found that Zfp143 mRNA was downregulated in differentiated C2C12 cells (Figure 3). Furthermore, an interesting inverse correlation between ZFP143 and *circPde4dip* expression was observed during myogenesis and circRNA silencing (Figure 4). These findings suggest that *circPde4dip* interacts directly with *Zfp143* mRNA, potentially leading to mRNA destabilization during the differentiation process. Notably, the circRNA of interest, *circPde4dip*, was found to bind to the 5’ UTR of *Zfp143* mRNA specifically. The binding of circRNA to the 5’-UTR could obstruct its crucial role in ribosome recruitment, leading to stalled translation and subsequent mRNA decay. Alternatively, circRNA binding might induce secondary structure alterations in the 5’ UTR, thereby exposing the mRNA to stress-induced degradation. Additionally, the competitive binding of circRNAs in the 5’ UTR could potentially disrupt the association of the cap-binding complex (CBC) and reduce mRNA stability. *Zfp143* mRNA has been previously reported to maintain the pluripotency in mouse embryonic stem cells [12] and is a major regulator in rat vascular smooth muscle cells [13]. As ZFP143 promotes stemness and cell proliferation, silencing *circPde4dip* reduced myotube formation by upregulating ZFP143. Together, we hypothesized that *circPde4dip* inhibits ZFP143 expression by directly interacting with the mRNA. During differentiation, upregulated *circPde4dip* interacted with *Zfp143* mRNA, destabilizing *Zfp143* mRNA and suppressing ZFP143 expression, ultimately favouring myotube formation (Figure 4).

Despite our work confirming the direct regulation of gene expression by circRNA-mRNA interactions in skeletal muscle, there are a few considerable limitations. First, the number and types of circRNAs may differ depending on the computational pipeline and sequencing depth used for circRNA identification. Second, the positions of circRNA – mRNA interactions were computationally identified. Third, considering that circRNAs and mRNAs interact with RBPs as well as miRNAs, whether the interacting sequences are free to interact with each other and which RBPs can indirectly regulate target mRNAs require further experimental validation. In addition, novel/unknown miRNAs interacting with *circPde4dip* may regulate myogenesis via an unknown regulatory axis that warrants further investigation. Fourth, the *in vivo* secondary and tertiary structures of interacting RNAs in endogenous cellular conditions may need to be considered when dissecting the functional significance of circRNA-mRNA hybrids. While these circRNA-mRNA mechanisms offer intriguing possibilities, an in-depth study of this regulatory mechanism can help us broaden our understanding of the mRNA-interacting circRNAs in myogenesis and other cellular events. Our study not only identified novel links between hundreds of circRNAs and mRNAs but also highlighted the need for follow-up work that offers considerable scope for the development of new RNA-based treatments.

## Experimental procedures

### Cell culture and differentiation

Mouse C2C12 myoblast cells were cultivated in a growth medium consisting of Dulbecco’s Modified Eagle’s Medium (DMEM, Gibco, 11,965,118) with Fetal Bovine Serum (FBS, Gibco, 10,270,106) of 15% concentration and penicillin-streptomycin (1%) (Gibco, 15,140,122). The growth medium of sub-confluent myoblast cells was replaced with a differentiation medium containing DMEM supplemented with 2% horse serum (Gibco, 26,050,070) and 1% penicillin-streptomycin [14]. The cells were supplemented with differentiation media for up to 6 days depending on the requirement, and differentiation was visualized under a phase-contrast microscope to monitor the formation of multinucleated elongated myotubes.

### AMT-mediated proximity ligation and RNA sequencing

The 4′-aminomethyltrioxsalen hydrochloride (AMT) (Sigma, A 4330), a compound known for its ability to induce RNA-RNA crosslinking, was diluted in DMEM and 2X phosphate-buffered saline to obtain a working concentration of 20 μg/ml, which was used as the irradiation medium. C2C12 myoblast cells and 6-days differentiated C2C12 myotubes were placed in irradiation media and then incubated at 37°C for 30 min [15]. UV crosslinking was then conducted at 365 nm to facilitate RNA-RNA crosslinking [16].

Total RNA was isolated from AMT-treated cells, followed by quality check using NanoDrop 2000, Multiskan Sky, or Qubit 4. Subsequently, the mRNA fraction was purified from 1 μg of the total RNA using the NEBNext® poly(A) mRNA Magnetic Isolation Module (NEB, E3370), followed by concentration and quality checks. The mRNAs and its interacting RNA complexes pulled down from the C2C12 cells were used for Crosslinking-Immunoprecipitation-Sequencing (CLiPP-Seq). The cDNA libraries were prepared using the NEB NeXT Ultra II Library Prep Kit (NEB, E7765S) with minor modifications to incorporate additional steps of de-crosslinking at 254 nm after RNA fragmentation [15]. The cDNA libraries were then analysed on TapeStation (Agilent, 5067–5582), followed by 150 bp paired-end sequencing on the Illumina HiseqX platform.

### RNA sequencing and BLAST analysis

The adapters from the RNA-seq reads were removed using the Cutadapt tool, and quality was checked by Fastqc, followed by alignment with the mouse reference genome assembly release of mm10. For circRNA identification, reads were aligned using the STAR aligner using the ChimSegmentMin-10 parameter of the CIRCexplorer2 pipeline [17]. The mature sequences of the circRNAs were used to perform BLAST against the mouse transcript (NCBI BLAST), as well as command-line BLAST, and only Plus/Minus interactions were filtered to identify the circRNA-mRNA hybrids.

### RNA isolation, RT-PCR, Sanger sequencing, and quantitative (q)PCR

Total RNA was extracted from 2-days differentiated C2C12 cells using either the TRIzol (Thermo Fisher Scientific, 15,596,018) or the MagSure All RNA isolation kit (RNA Biotech). cDNA synthesis with or without RT enzyme (± RT) was performed from total RNA using High Capacity (Thermo, 4,368,814) or Maxima Reverse Transcription Kit (Thermo, EP0743). The RT/no-RT cDNA samples were then amplified with divergent primers, using 2X DreamTaq PCR master mix (Thermo, A25778), followed by visualization of the PCR amplicons on a 2% SYBR gold (Thermo, S11494) stained agarose gel. For backsplice junction sequence validation, RT-PCR products were purified using Purelink PCR Purification kit (Thermo, K310002) and subjected to Sanger sequencing using one of the divergent primers [18]. For RT-qPCR, 2X PowerUP SYBR Green Master Mix (Thermo, A25778) was used, and target enrichment was calculated using the delta-CT method, considering *18s* rRNA or *Gapdh* mRNA as internal controls [19].

### RNase R treatment and stability assay of circRNA & mRNA

For RNase R treatment assay, total RNA was isolated from 2-days differentiated C2C12 cells; 5 μg of total RNA was treated with or without 0.5 μL of RNase R (Lucigen, RNR07250) at 37°C for 60 min, followed by RNA isolation and cDNA synthesis. RT-qPCR analysis was performed on mock and RNase R-treated samples using specific primers for linear and circRNAs (Supplementary Table S3) [20]. To check the stability of circRNAs over mRNAs, 2-days differentiated C2C12 cells were treated with actinomycin D (Sigma, A4262) at a working concentration of 10 μg/mL for 0 h, 0.5, 1, 2, 4, and 8 h, followed by total RNA isolation, cDNA synthesis, and RT-qPCR analysis of *circPde4dip* and *c-Myc* expression levels at different time points.

### Poly(A) RNA and circRNA pulldown assays

For mRNA pulldown, 10 μg of the total RNA isolated from 2-days differentiated C2C12 cells was subjected to poly(A) pulldown using NEBNext® Poly(A) mRNA Magnetic Isolation Module following the manufacturer’s instructions. For native mRNA pulldown assay, 2-days differentiated C2C12 cells were lysed with polysome extraction buffer (PEB), followed by incubation with NEBNext Oligo d(T)25 Beads in 1X TENT buffer for 90 min at 4°C in a rotator. The beads were then washed with the wash buffer, followed by elution of mRNA in 12 μl of elution buffer. One μg of total RNA was used as an input control, and a total 12 μl of eluted mRNA was used for cDNA synthesis and RT-qPCR analysis of mRNA and circRNAs in pulldown samples compared to input samples [9]. circRNA pulldown was performed using biotin-labelled antisense oligo (ASO), as described previously [21]. Briefly, 3-days differentiated C2C12 cells were lysed with polysome extraction buffer (PEB). Subsequently, the supernatant was collected and equally distributed for pulldown with control-ASO or *circPde4dip*-ASO. An equal volume of (2X) TENT buffer was then added to the supernatant and subjected to hybridization using 1 μl of 100 μM control-ASO and *circPde4dip*-ASO, followed by ASO-complex pulldown using streptavidin beads. Both control and circRNA pull-down samples were used for cDNA synthesis and RT-qPCR analysis.

### circPde4dip silencing, cell proliferation, and western blotting

For circRNA silencing, antisense GapmeR oligos targeting the unique backsplice junction sequence of circRNAs were synthesized. Actively growing C2C12 cells were subjected to GapmeR LNA transfection with 100 nM twice at 24 h intervals using Lipofectamine RNAiMAX (Invitrogen, 13,778,150) and allowed to differentiate for 72 h. Total RNA was isolated, followed by cDNA synthesis and RT-qPCR analysis to determine the expression of circRNA and target mRNA. Using the same experimental design, total cellular protein was purified 72 hours post-transfection using 1X RIPA buffer (Himedia, TCL131). Protein concentrations were estimated using Pierce BCA Protein Assay Kit (Thermo, 23,227) and then resolved in 10% SDS-resolving gel. Western blotting was performed to check the expression of ZFP143 protein (Invitrogen, PA5116147), and normalized to HSP90 protein (CST, 4877S) expression. For cell proliferation assay, the C2C12 cell numbers in control and *circPde4dip*-silenced cells were counted after 2 days of *circPde4dip* silencing using the Countess 3 Automated Cell Counter (Thermo).

### Statistical analyses and visualization

In the present study, Student’s t-test was employed to calculate the statistical significance of the observed findings, with a threshold of *p < 0.05, as statistically significant. All experimental results and figures were obtained from at least three independent biological replicates, ensuring the reliability and robustness of the results. For data visualization and analysis, various software tools were utilized, including Microsoft Excel, GraphPad Prism, Cytoscape, and R studio.

## Supplementary Material

Supplementary Figures_Revised_13 Oct.docx

## Data Availability

All the data generated in this study are included in the main text or supplementary data. The pre-print version of this manuscript is available in BioRxiv. The RNA-seq data generated in this study were deposited in the Indian Biological Data Centre (IBDC) with INSDC Project Accession number PRJEB90311/ERP173326. The Supplementary Tables can be found in the Figshare data repository (https://figshare.com/s/92d62b495736fe07100f).

## Pre-print statement

A pre-print version of this manuscript is available on the bioRxiv (https://www.biorxiv.org/cgi/content/short/2025.09.17.676975v1) [22].

## Highlights

- Identified mRNA-associated circRNAs in differentiating C2C12 myoblasts.
- The first report identifying the regulation of myogenesis by circRNA-mRNA interaction.
- *circPde4dip* regulates C2C12 differentiation by interacting with *Zfp143* mRNA
