# Supplementary material for: Circular RNA Pde4dip regulates myogenesis by interacting with Zfp143 mRNA: a novel regulatory axis
Source: RNA Biol. 2025 Oct 31;22(1):1–11. doi: 10.1080/15476286.2025.2583576 (PMC12591587; doi:10.1080/15476286.2025.2583576)

**Circular RNA *Pde4dip* regulates myogenesis by interacting with *Zfp143* mRNA: A novel regulatory axis**

Suman Singh, Arundhati Das, Gaurahari Sahoo, Amaresh Chandra Panda*

*Correspondence: Amaresh C. Panda; Email: [amaresh.panda@ils.res.in](mailto:amaresh.panda@ils.res.in)

Institute of Life Sciences, Nalco Square, Bhubaneswar, Odisha, India

**SUPPLEMENTARY TABLES AND FIGURES**

**Supplementary Table S1:** CircRNA annotation of C2C12-seq

**Supplementary Table S2:** CircRNA-mRNA BLAST analysis in C2C12 cells

**Supplementary Table S3:** Oligonucleotide


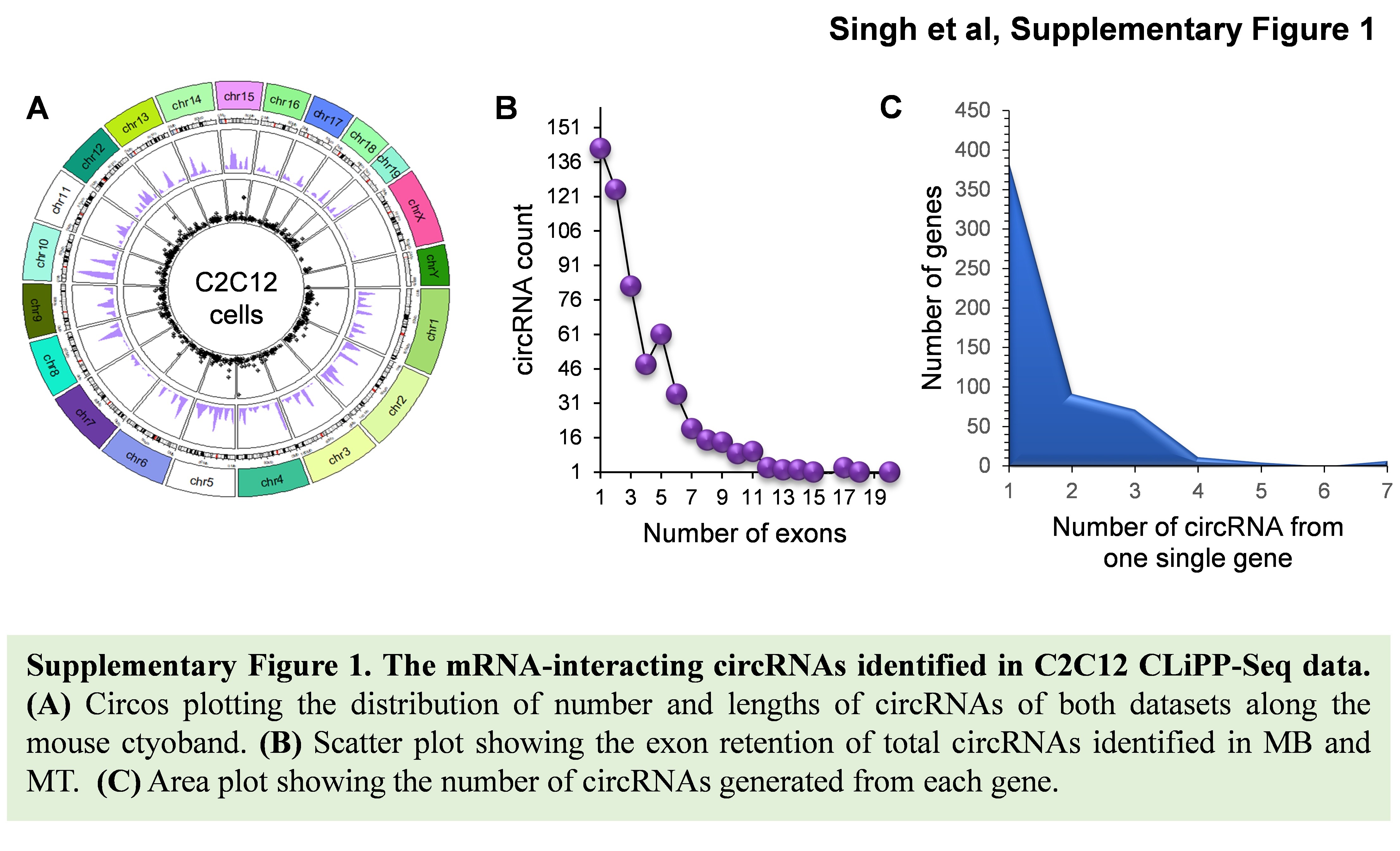

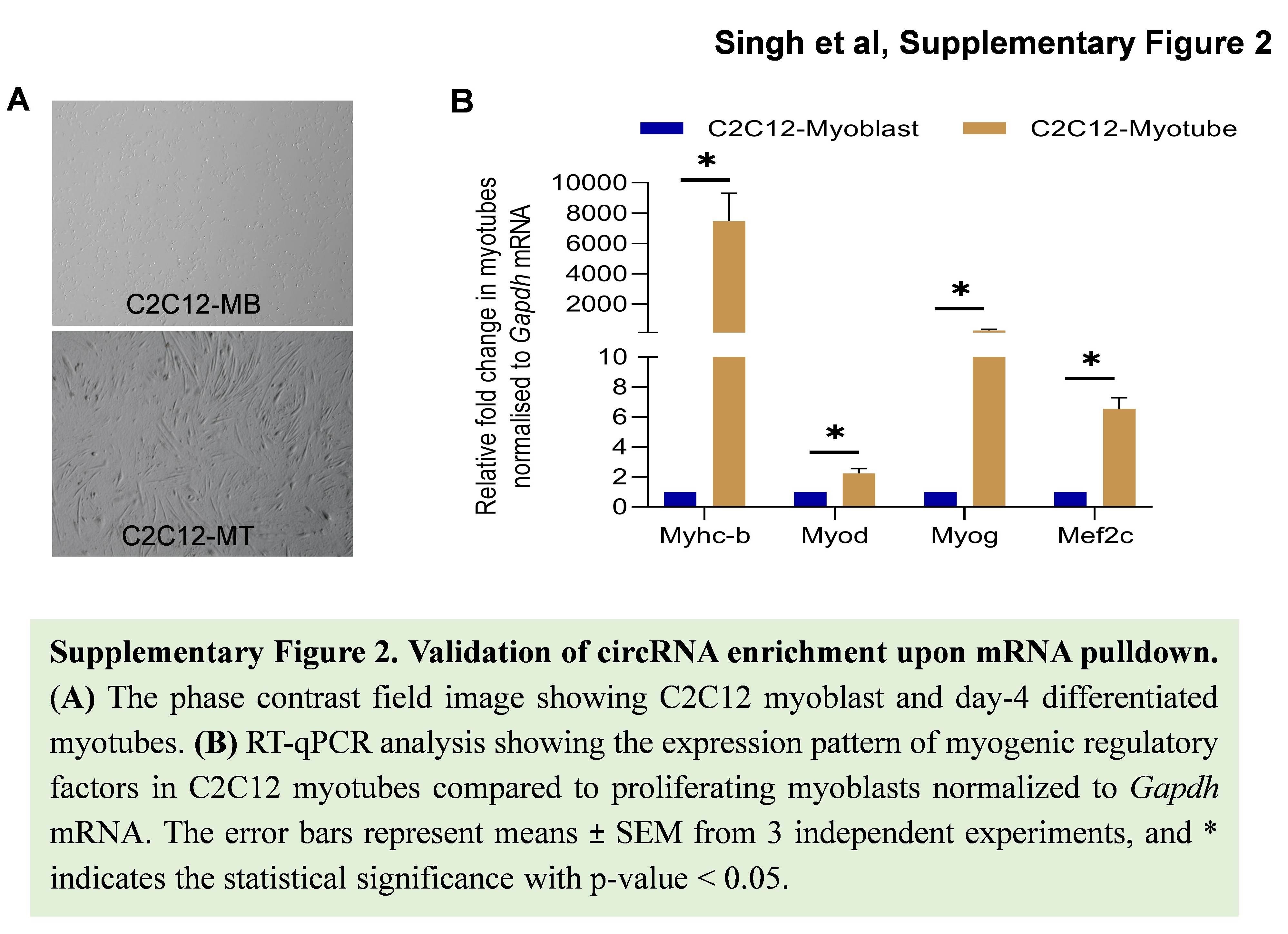

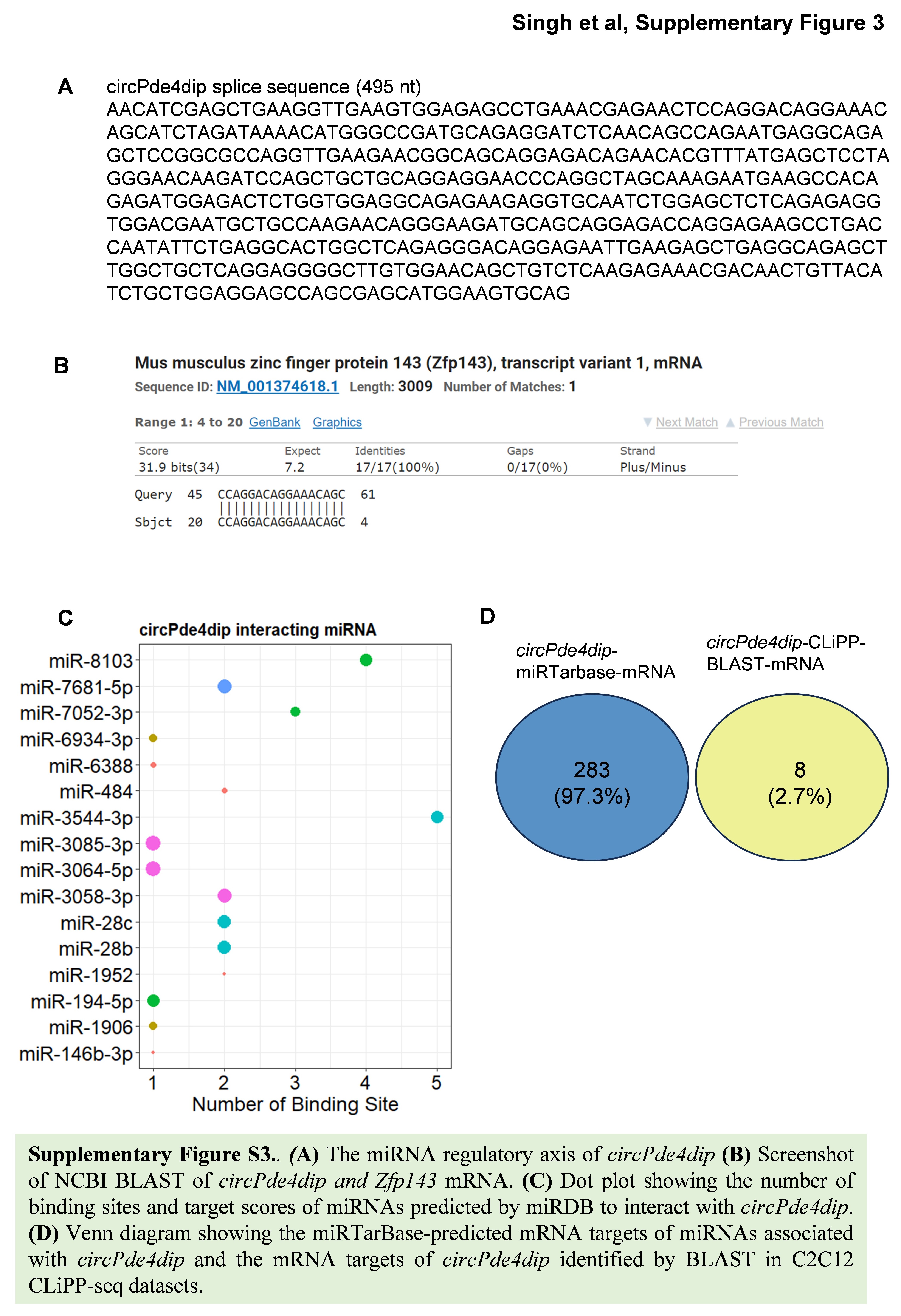

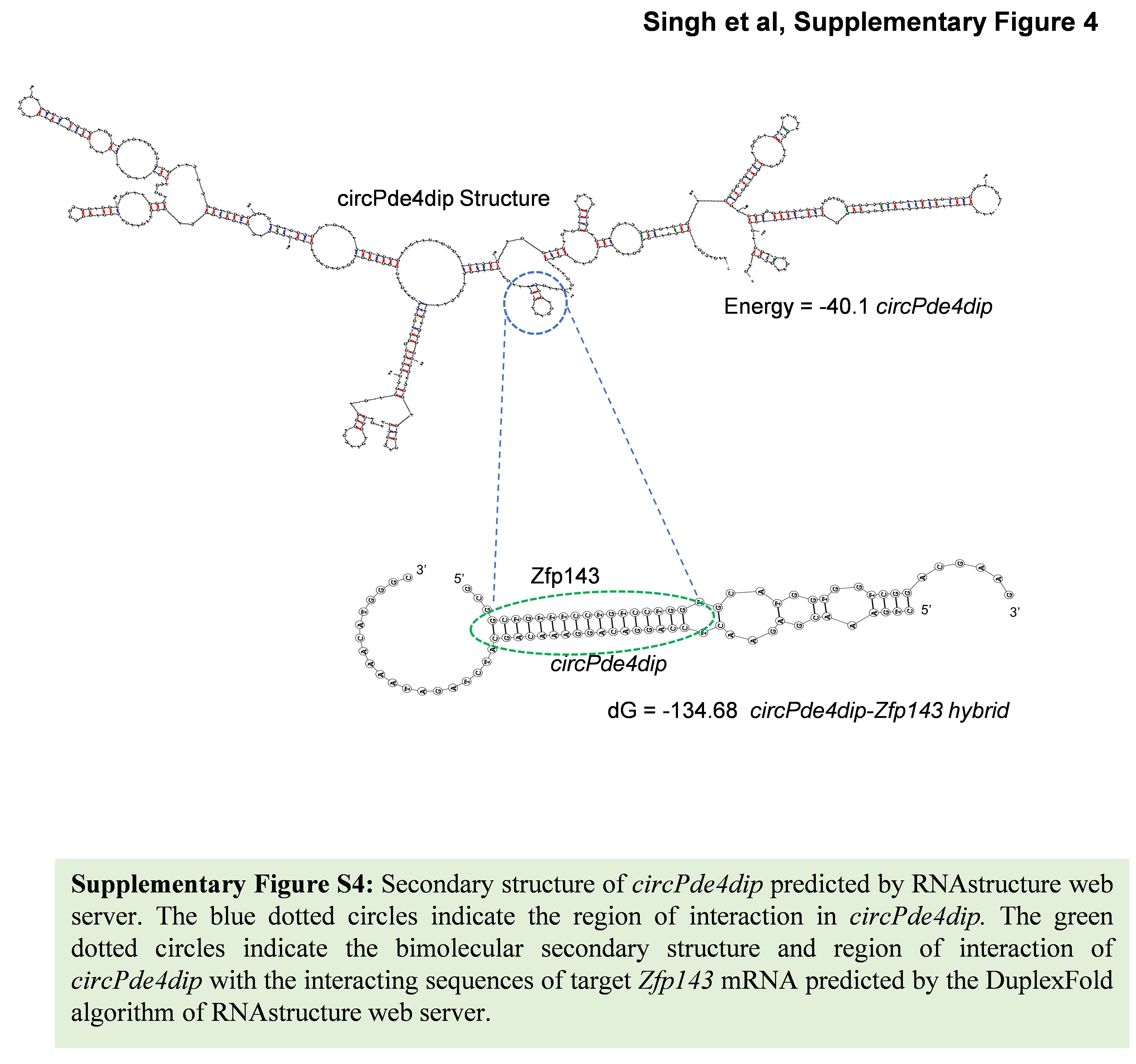

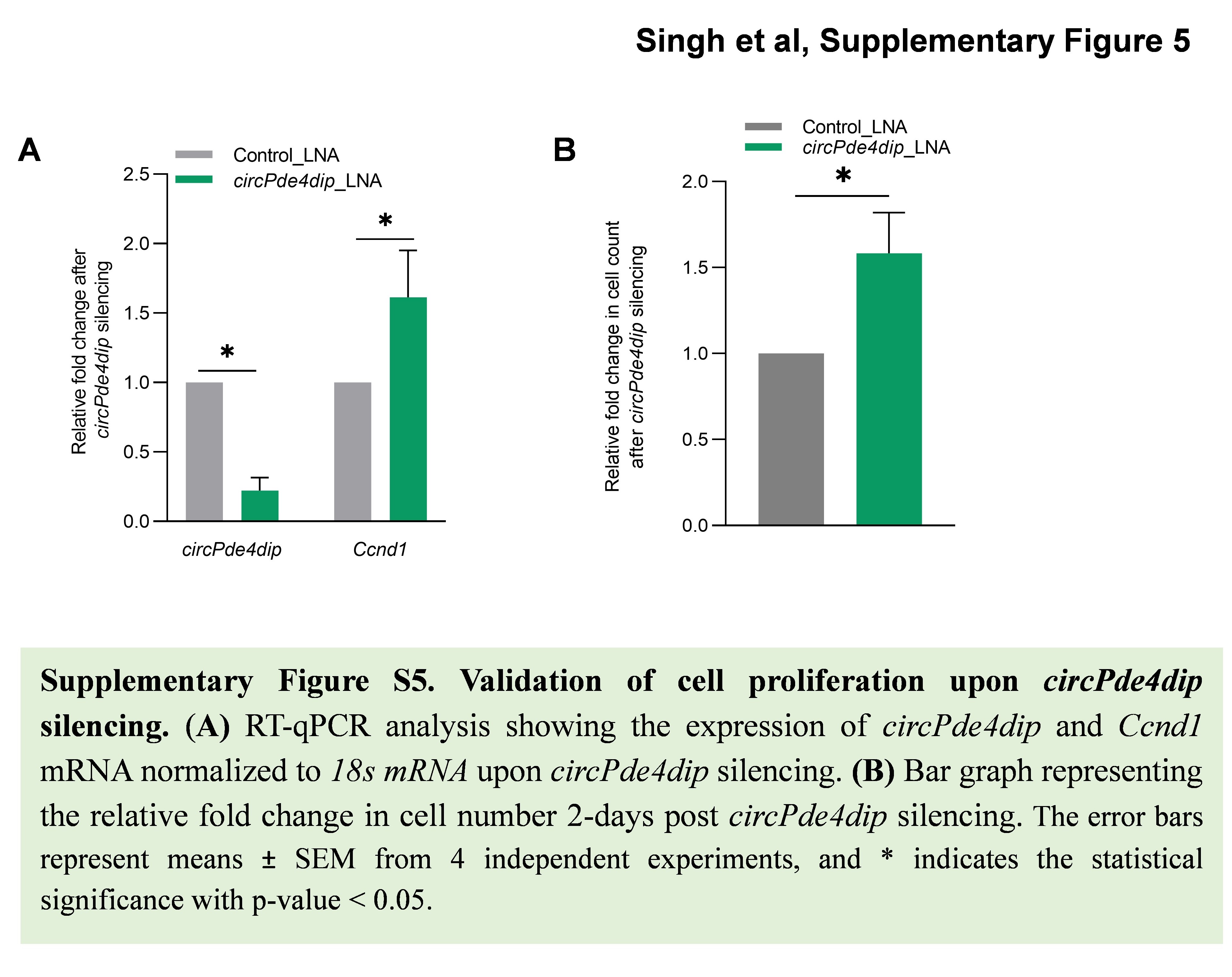

Supplement: Supplementary Figures_Revised_13 Oct.docx [file KRNB_A_2583576_SM5467.docx]
